# Large-Area Deterministic Stamping of 2D Materials on Patterned Surfaces

**DOI:** 10.1021/acsnano.6c04231

**Published:** 2026-07-12

**Authors:** Bernardo S. Dias, Reynolds Dziobek-Garrett, Gabriella Mentasti, Abhishek Gupta, Alexander Lambertz, Esther Alarcón-Lladó, Peter Schall, Roland Bliem, Jorik van de Groep

**Affiliations:** † Van der Waals-Zeeman Institute, Institute of Physics, University of Amsterdam, Amsterdam 1098 XH, The Netherlands; ‡ 530573 Advanced Research Center for Nanolithography, Science Park 106, Amsterdam 1098 XG, The Netherlands; § Center for Nanophotonics, 55952 AMOLF, Amsterdam 1098 XG, The Netherlands; ∥ van ‘t Hoff Institute for Molecular Sciences, 1234 Universiteit van Amsterdam, Amsterdam 1098 XH, The Netherlands

**Keywords:** 2D materials, large-area transfer, deterministic stamping, photonic metasurfaces, heterostructures, mechanical exfoliation

## Abstract

2D materials and their monolayers have attracted widespread interest by virtue of their unique electronic and optical properties. In addition to their remarkable physical characteristics, their atomically thin nature enables integration in ultracompact photonic and electronic devices, with potential for dynamic tunability via strain, charge carrier modulation or heterostructure engineering. While early research relied on micrometer-scale mechanically exfoliated flakes, recent advances—particularly gold-assisted exfoliation of transition metal dichalcogenides (TMDCs)—have enabled the preparation of high-quality, large-area monolayers, facilitating scalable device integration. For the field of nanophotonics in particular, the ability to transfer large-area 2D materials onto both flat and patterned substrates is essential for the development of functional devices. However, existing transfer techniques are often limited in scalability, compatibility with structured surfaces, or preservation of material quality. Here, we present a versatile and reliable transfer method of large-area monolayers and hBN/monolayer heterostructures onto both flat and nanostructured substrates. Our approach, based on the physical properties of low-density polyethylene, enhances excitonic emission of TMDCs and is compatible with a variety of device architectures. We demonstrate its applicability by fabricating devices that modulate the photoluminescence of TMDC monolayers through the manipulation of the photonic environment, strain or electrical gating. We further demonstrate the fabrication of van der Waals heterostructures using the same method. By enabling clean transfer of a wide range of monolayers and heterostructures, this technique offers a practical pathway for the development of next-generation optoelectronic platforms with improved functionality, scalability, and tunability.

## Graphical abstract

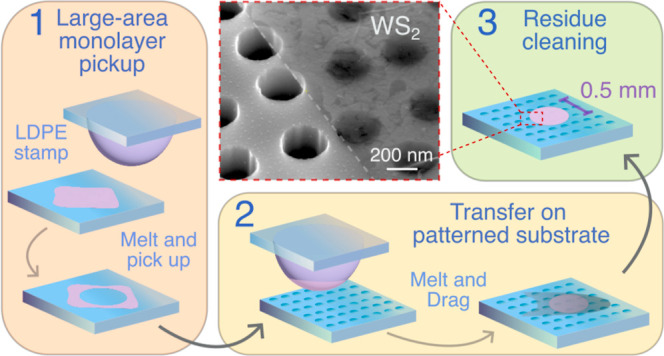

The development of 2D van der Waals (vdW) material fabrication and exfoliation procedures have enabled unprecedented control of their layered structure, leading to breakthroughs in both the fundamental study of 2D material physics and the development of novel devices to manipulate charge carriers,^1^ light,^2^ or spin waves^3^ at the nanoscale. The vast range of 2D materials available^4^ has unveiled remarkable new material properties including large carrier mobility,^5^ bandgap tunability,^6^ and high thermal conductivity.^7^ Moreover, the ability to stack different materials in heterostructures to explore new phenomena including Moiré physics,^8^ topological effects,^9^ and interlayer excitons,^10^ further expands the applicability of vdW materials. In addition to the unique intrinsic properties of 2D materials and their heterostructures, engineered interactions with the underlying substrate can be leveraged to further tune the material’s properties. For example, monolayer transition metal dichalcogenides (TMDCs) in contact with insulating materials support strongly bound excitons, which can be observed through bright photoluminescence (PL), while contact with metals can quench the emission.^11,12^ Alternatively, (nano)­patterned substrates have been employed to induce strain in monolayer TMDCs, leading to single photon emission from defect states,^13,14^ and the integration of such monolayers with optical metasurfaces^15^ has resulted in the observation of strong light–matter interactions, such as exciton polariton and plexitonic states.^16,17^ The strong and tunable excitonic light–matter interaction has already enabled novel applications such as optical modulators,^18,19^ beam steering,^20^ and photodetectors.^21^ These exciting advances highlight the importance of the development of techniques that offer controllable stacking of these thin layers and integrate them with a wide variety of substrates.

While initial 2D material-based devices have shown very promising demonstrations that highlight the unique capabilities of these materials, they largely rely on small-area, mechanically exfoliated flakes. In this regard, several techniques have been developed to perform dry-transfer of these small flakes, relying on polymers such as PDMS,^22^ PVC^23^ or PPC,^24^ or Si_3_N_4_ membranes.^25^ Additional methods that directly combine these monolayers with hBN are readily proposed, with the notable example of the hot pickup technique, which capitalizes on the stronger 2D material vdW attraction to pick up monolayers with the precise dimensions of the top hBN flake.^26^

Recently, the development of the gold-assisted exfoliation (GAE) technique has marked a crucial advance in 2D material handling by enabling the exfoliation of TMDC monolayers up to centimeter scale, with material qualities comparable to mechanically exfoliated layers.^27–29^ At the same time, transferring these large and high-quality monolayers for the fabrication of large-area nanophotonic and electronic devices remains a critical challenge. In addition to the size limitation, existing transfer methods crucially depend on the reliable adhesion of the monolayer to the target substrate, which makes the transfer onto patterned surfaces very challenging. To mitigate this dependence, other methods have been devised where a small sacrificial layer of the polymer is transferred along with the heterostructure onto a flat surface, which is removed afterward using a solvent.^30^ Despite these advances, the ability to transfer large-area 2D materials onto patterned substrates with minimal surface adhesion remains a major outstanding challenge. At the same time, such ability would open a vast range of opportunities where high-quality 2D materials are integrated in large-area devices such as high-Q metasurfaces, array-based photodetectors, and flexible optoelectronic platforms.

Here, we demonstrate a simple method that allows reliable transfer of both large-area monolayers and hBN/monolayer heterostructures to patterned or unpatterned substrates, ranging from flat to high-aspect ratio and low adhesion patterned interfaces. In this process, we use low-density polyethylene (LDPE), an inexpensive and readily available polymer with advantageous properties such as low melting temperature and tunable surface energy.^31,32^ By assembling and characterizing devices such as angularly selective metasurfaces, strain-engineered emitters, and electrically gated TMDC monolayers, we show how the technique facilitates the development of advanced 2D optoelectronic platforms. We also highlight how this transfer method enables the exploration of 2D material physics such as interlayer exciton formation over large areas, offering new possibilities for further fundamental studies of these materials.

## Results

### Transfer of 2D Material Monolayers

To analyze the proposed method without influence of substrate effects, we demonstrate the transfer of a GAE large-area WS_2_ monolayer from a SiO_2_ substrate to another target SiO_2_ substrate (Figure 1). We break the procedure into three discrete steps: the pickup, the transfer, and stamp residue removal.

**Figure 1:**
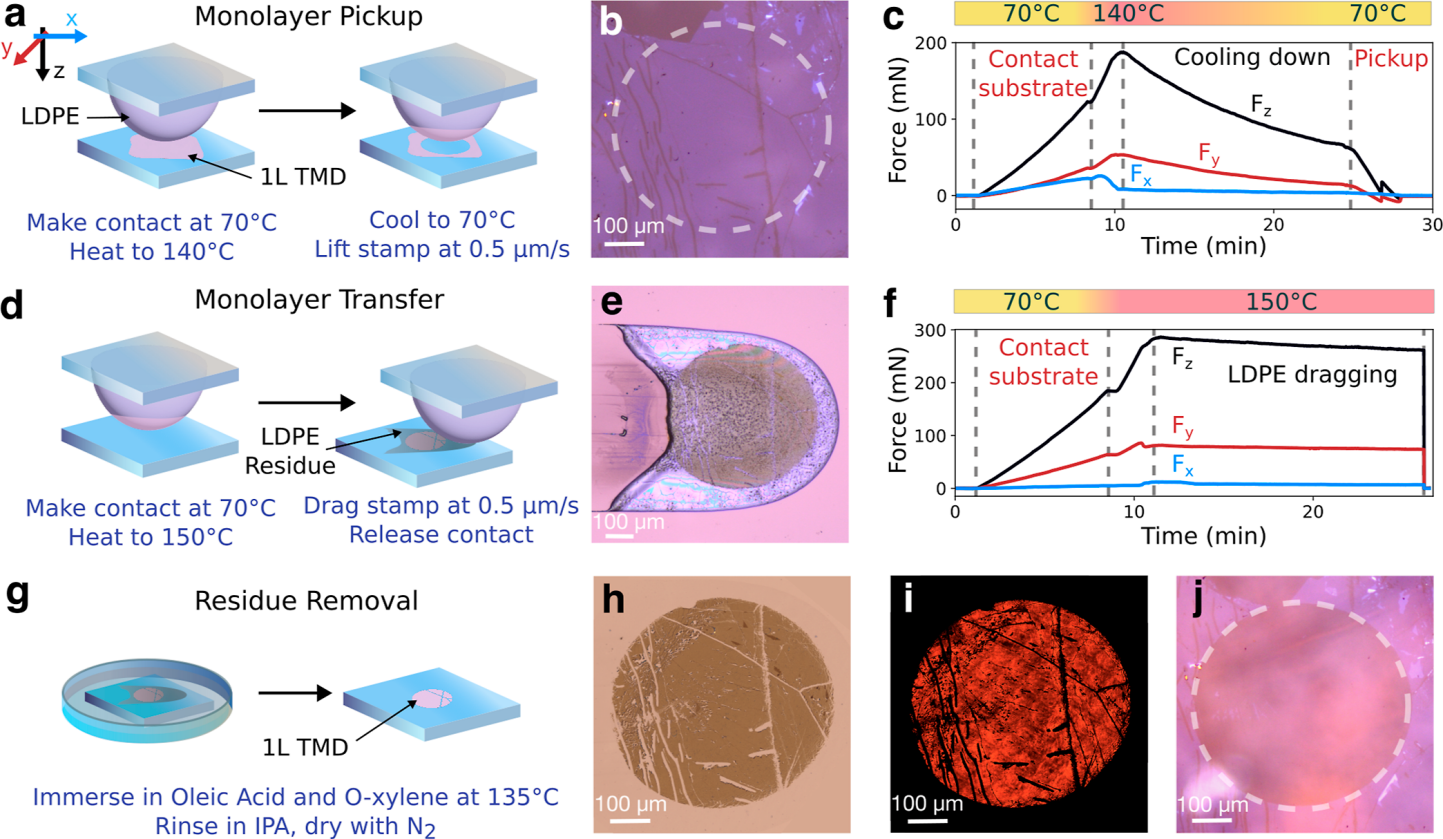
Transfer method for large-area monolayers using the LDPE-based stamp. (a) Procedure for monolayer pickup. (b) Bright field image of the WS_2_ monolayer after GAE, before pickup. The cracks in the monolayer originate from the exfoliated bulk crystal. (c) Normal and in-plane force measurements during the pickup procedure. (d) Procedure for monolayer transfer. (e) Monolayer and LDPE residue after transfer to the target substrate. (f) Normal and in-plane force measurements during the transfer of the monolayer. (g) Procedure for LDPE residue removal. Bright field (h) and wide field PL (i) image of the transferred WS_2_ monolayer. The grid-like structure with darker regions are stitching artifacts resulting from the merger of multiple high resolution microscope images into a large-area overview. (j) Bright field image of the initial substrate after transfer, highlighting the original transferred area.

The transfer method relies on the fabrication of a stamp composed of a heat-resistant superglue half-sphere covered by LDPE cling film (Figure 1a, see Supporting Information Section 1 for details on the fabrication of the stamp), which enables precise and selective pickup of 2D materials without contaminating other regions of the sample.^23,33^ To allow deterministic pickup of a 2D material monolayer—where the monolayer is placed in a controlled and reproducible position—the stamp is mounted on an *xyz*-micrometer precision stage and approaches the GAE monolayer (Figure 1b) at a speed of 0.5 μm/s to make slow contact. The monolayer is placed on a temperature controlled holder, set to 70 °C. We use force sensors in the stage to measure the forces involved during the stamping procedure (see Supporting Information Section 2) both in the plane of the 2D material (*F*_*x*_ and *F*_*y*_) and perpendicular to it (*F*_*z*_, Figure 1c). This not only offers better control and repeatability of the transfer, it also provides crucial information on the contact and friction dynamics throughout the process. During initial contact with the substrate, the normal force (*F*_*z*_) increases to 120 mN, at which point we stop the approach. The in-plane force components also increase slightly due to a small tilt of the glass slide and coupling between *F*_*z*_ and *F*_*y*_ caused by the stamping tool geometry (see Supporting Information Section 2). After the stamp has made contact with the monolayer, the system is heated to 140 °C inducing a phase transition in which the LDPE melts, achieving conformal contact with the monolayer. This crucial step gives strong adhesion of the monolayer to the stamp and underpins the large yield in the pickup process. As a result of thermal expansion during the phase transition, we see a rapid increase in *F*_*z*_ (Figure 1c). The system is then cooled down to 70 °C, leading to LDPE solidification and a decrease in forces due to thermal contraction. We can then pick up the monolayer with a vertical motion at 0.5 μm/s. Here, the spikes in the associated force curves indicate some intermittency in the detachment of the polymer due to adhesive forces.^34^

Second, we discuss the monolayer transfer, in this example to a new SiO_2_ substrate, as illustrated in Figure 1d–f. We make contact with the substrate with the same parameters as for the pickup (0.5 μm/s, 70 °C), with Figure 1f showing an initial force curve similar to Figure 1c. The system is then heated to 150 °C (rather than 140 °C), further melting the LDPE and considerably reducing its viscosity compared to at 140 °C.^31^ Next, we slide the stamp parallel to the substrate interface along the *y*-axis away from the original contact area, leaving behind the monolayer covered by a sacrificial layer of LDPE (Figure 1e). During the onset of dragging, we observe the transition from static to dynamic friction, marked by an oscillatory behavior in *F*_*y*_ (Figure 1f). The slow decrease in *F*_*z*_, indicates possible mass loss due to the transfer of LDPE to the substrate (Figure 1e). Finally, when the stamp is no longer in contact with the 2D material, we safely lift it from the substrate, which then slowly cools to room temperature.

Third, residual LDPE on the top side of the monolayer (Figure 1e) is removed by sequential immersion in two baths of oleic acid and two o-xylene baths (30 min each at 135 °C), followed by rinsing in isopropanol (IPA) and drying with N_2_ (Figure 1g). To assess the impact of the transfer procedure, we quantify the monolayer cracked area before transfer, which is 8% due to cracks in the bulk crystal, and after transfer, where it increases to 14%, mostly generated at the edge of the stamp contact area (Figure 1b,h). As such, the transfer procedure only modestly increases the cracked area fraction and largely preserves the monolayer topography, while the material sustains its excitonic PL that is characteristic for WS_2_ monolayers (Figure 1i). After transfer, the original substrate is left with a clean area, where 99.5% of the monolayer that was in contact with the stamp was picked up (Figure 1j, inside the white circle). We emphasize that while Figure 1 shows the transfer of a monolayer ∼0.5 mm in diameter, the size is only limited by the geometry of the monolayer after the GAE process and the controllable contact area between the stamp and the monolayer, which enables both smaller and larger areas to be transferred.

By using the same material (SiO_2_) for both the source and target substrate, we can directly monitor the impact of the transfer on the material quality. First, to assess possible transfer-induced strain, we measure Raman spectra of the monolayer during multiple stages of the procedure (Figure 2a). We observe a considerable redshift of the *E*_2*g*_^1^ peak when WS_2_ is exfoliated from the bulk crystal during the initial GAE step (354 cm^–1^, measured on Au), in agreement with recent literature that argues that GAE is mediated by biaxial strain induced in the monolayer, decreasing the bond strength between the first and second layers.^35^ Comparing the same *E*_2*g*_^1^ peak for monolayer WS_2_ on quartz before pickup (356 cm^–1^) and after transfer and LDPE cleaning a smaller shift is observed, which we argue is mostly caused by changes in carrier doping rather than strain (see Supporting Information Section 3 for a detailed discussion, Figure S4a,b). As such, we conclude that the transfer process does not induce a significant strain in the monolayer.

**Figure 2:**
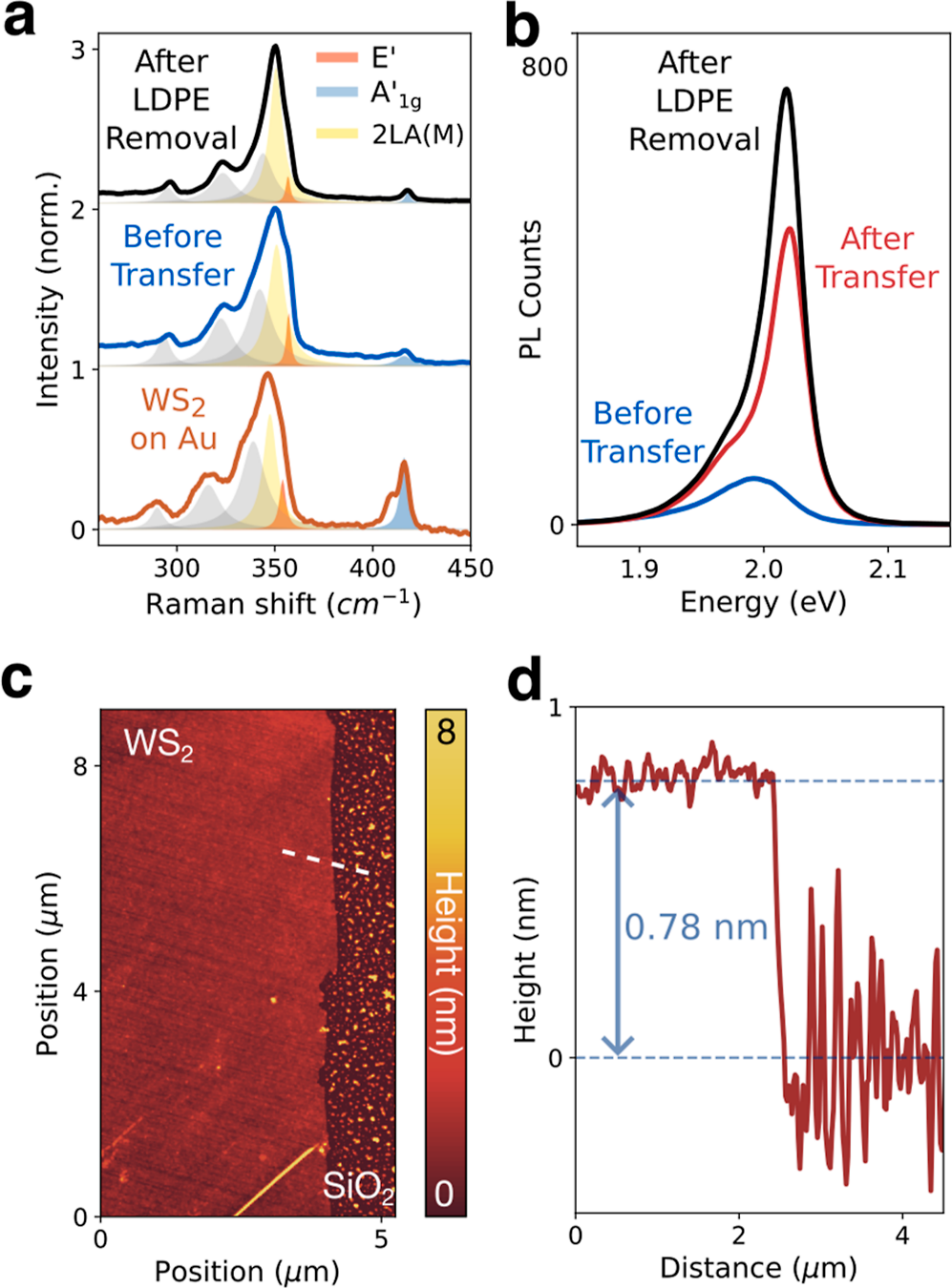
Characterization of the monolayer before and after transfer. (a) Raman and (b) PL spectra of the monolayer before and after transfer, as well as after LDPE residue removal. (c) AFM map and (d) averaged line scan of the monolayer, showing the thickness after transfer. The white dashed line in (c) highlights the central location where the line scan in (d) is taken.

Two crucial observables that characterize the quality of monolayer TMDCs for applications in photonic devices are the PL line width and intensity. Analogous to the Raman analysis, we study the excitonic PL spectra at different stages of the transfer process (Figure 2b). Compared to the as-exfoliated film (blue), the LDPE transfer induces a marked improvement in the PL by transitioning from a spectrum dominated by emission from trions to neutral excitons. Here, the exciton line width decreases from 38 to 26 meV, combined with a strong enhancement of the PL emission intensity (red). These LDPE-induced changes are unexpected and are not previously reported in the literature. Combining analysis of both Raman and PL data, we propose that the PL spectrum changes are induced by a variation in the doping level of the monolayer (see Supporting Information Section 3 for a detailed discussion). While the exact origin of this doping change is not clear, the biggest change comes from lifting the monolayer from its initial substrate, while simply encapsulating the monolayer in LDPE or washing with oleic acid does not induce the same blue shift and intensity enhancement (see SI Section 3, Figure S5). Lastly, the subsequent removal of the LDPE residue with oleic acid maintains the narrow line width and further increases the emission intensity, reaching a final enhancement over 19 times. Previous work using oleic acid on TMDCs suggests that the enhanced PL quantum yield results from (i) passivation of sulfur vacancies, reducing the density of trap states available; and (ii) protection of the monolayer from atmospheric reactants by the alkyl tail of the oleic acid encapsulating the surface.^36,37^

Finally, to explore any polymer residue on the surface of the monolayer leftover from the transfer process, we perform atomic-force microscopy (AFM) scans on the monolayer after transfer (Figure 2c,d). A small-scale residue forms on the substrate surface, which Raman analysis identifies as remnants of the LDPE layer, similar to the residual transfer contamination observed in other dry-transfer techniques.^38^ Interestingly, this residue is mostly absent on the monolayer, possibly due to surface energy differences between the substrate and the 2D material. Taking a line section and averaging over the neighboring pixels, we verify a height step of 0.78 nm, showing absence of a layered residue on top of the monolayer. Overall, we conclude that the presented method not only allows for the clean transfer of the monolayer but simultaneously improves the exciton emission from the TMDC samples.

Besides flat surfaces, our proposed method also enables transfer to nanopatterned substrates, where the surface adhesion is smaller due to a reduced contact area. To illustrate this, we transfer a large WS_2_ monolayer onto a SiO_2_ (55 nm)/Si substrate patterned with nanoholes (covering 25% of the surface area) arranged in a hyperuniform pattern (Figure 3). We obtain a 0.18 mm^2^ covered area with uniform PL emission (Figure 3a,b). Using scanning-electron microscopy (SEM), we confirm that the monolayer dresses the nanopatterned surface with the material covering the nanoholes without rupturing (Figure 3c,d). To quantitatively assess the topography of the monolayer on top of the pattern, we perform AFM measurements and observe that the uncovered holes display a depth of approximately 120 nm, while the WS_2_-covered holes exhibit a very minor height profile due to draping of the monolayer over the hole (Figure 3e,f). By applying a parabolic fit to the monolayer over the nanohole gap (inset of Figure 3f, more information on Section 4 of Supporting Information), we calculate a strain of 4%, well below the threshold failure strain of 15 to 20% previously reported.^39^

**Figure 3:**
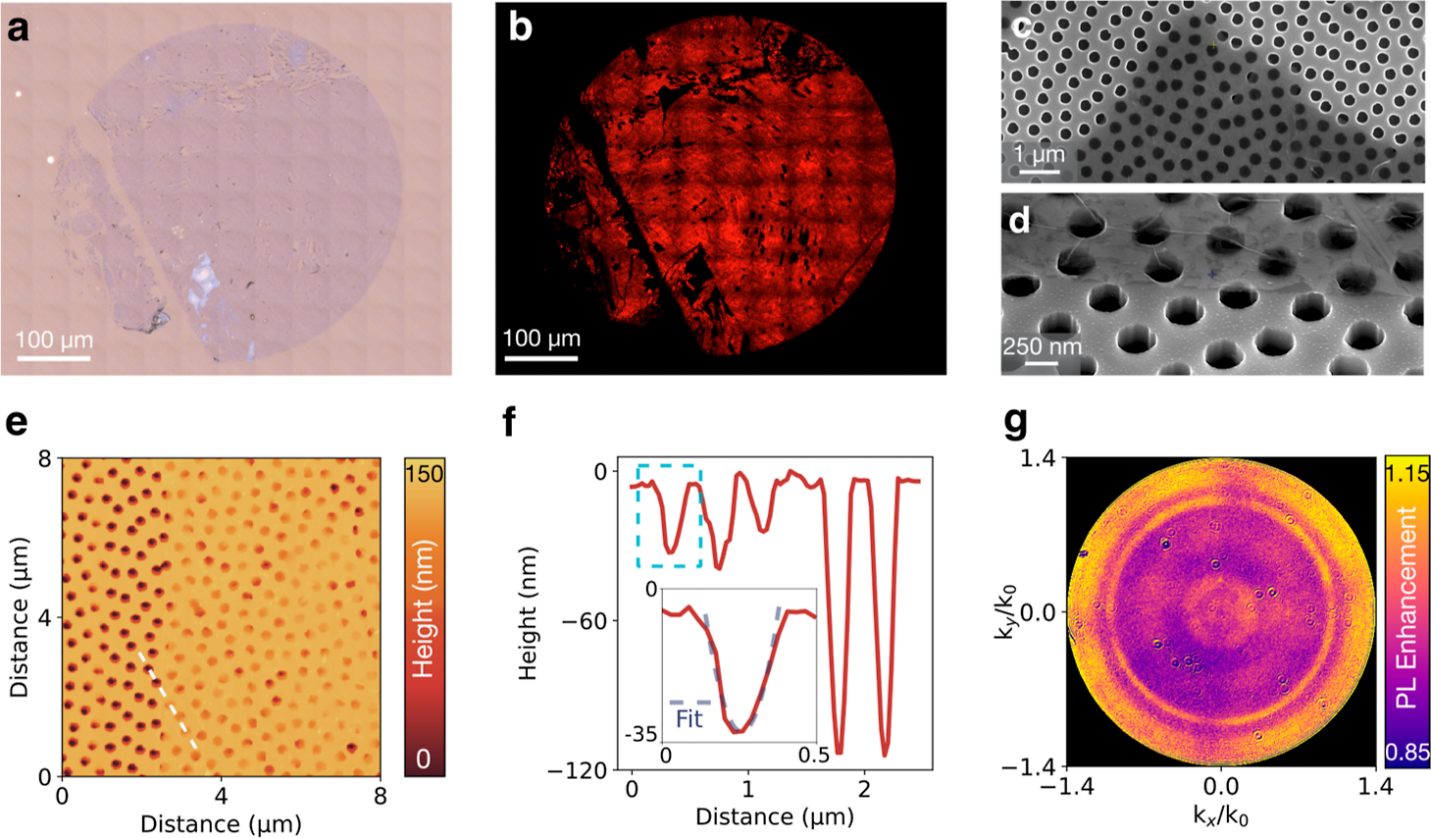
Transfer of a WS_2_ monolayer on a SiO_2_/Si substrate with hyperuniform patterned nanoholes. (a) Bright field and (b) wide field PL images of the transferred WS_2_ monolayer. Top view (c) and tilted (d) SEM images of the monolayer conforming to the substrate, where monolayer draping over the holes is clearly visible. AFM map (e) and cross section (f) of the monolayer on the patterned substrate. Inset: zoomed trace of a monolayer covered nanoholes (indicated in red dashed square) showing the parabolic fitting for strain calculation. (g) Back focal plane (BFP) microscope image of the PL emission of the sample, showing a ring with emission enhancement at *k*_*x*_/*k*_0_ = 1.016 due to light scattering by the hyperuniform pattern.

The transfer of 2D materials onto such large-area nanopatterns opens new opportunities for studying the interaction of delocalized photonic resonances with material resonances such as excitons. In this example, by placing a WS_2_ monolayer on top of a hyperuniform pattern, we demonstrate the ability of directional PL emission through modification of the photonic environment. An important characteristic of hyperuniform patterns is the suppression of the structure factor *S*(*k*) at *k* = 0, where *k* is the in-plane wavevector of light. This effect manifests itself as an absence of long-range density fluctuations while simultaneously showing a disordered local arrangement that remains statistically uniform over large scales, without periodicity.^40,41^ This suppression results in a redistribution of scattered light intensity to larger wavevectors, which in this case lie beyond the light line in air. Using normalized back focal plane (BFP) measurements with oil immersion, we measure a ring-like enhancement of the PL at *k*_*x*_/*k*_0_ = 1.016, corresponding to a wavevector of *k*_*x*_ = 10.3 μm^–1^ (Figure 3g, details on the normalization included in Supporting Information Section 5). At these wavevectors, we verify an emission enhancement up to 15%, while lower wavevectors show reduced emission, confirming the directionality of PL. From SEM images we also extract *S*(*k*) of the pattern (SI Section 5, Figure S6), verifying excellent agreement between the emission enhancement in the BFP and the peak in the structure factor. Altogether, this example demonstrates how our LDPE transfer technique offers opportunities for the merger of large-area 2D monolayers and photonic metasurfaces with decreased surface adhesion.

### Transfer of hBN/1L Heterostructures

One of the crucial features in 2D material research is the ability to stack different layers into heterostructures, enabling the assembly of novel materials that outperform the sum of their individual components. In particular, heterostructures combining hexagonal boron nitride (hBN) with TMDC or graphene monolayers have been employed extensively to provide atomically flat surfaces free of defects and charge traps. This improves the material’s carrier mobility and intrinsic optical and electronic properties of the monolayers.^42^ Here, we demonstrate how our LDPE transfer technique can be used to fabricate large-area hBN (top)/1L (bottom) heterostructures (Figure 4).

**Figure 4:**
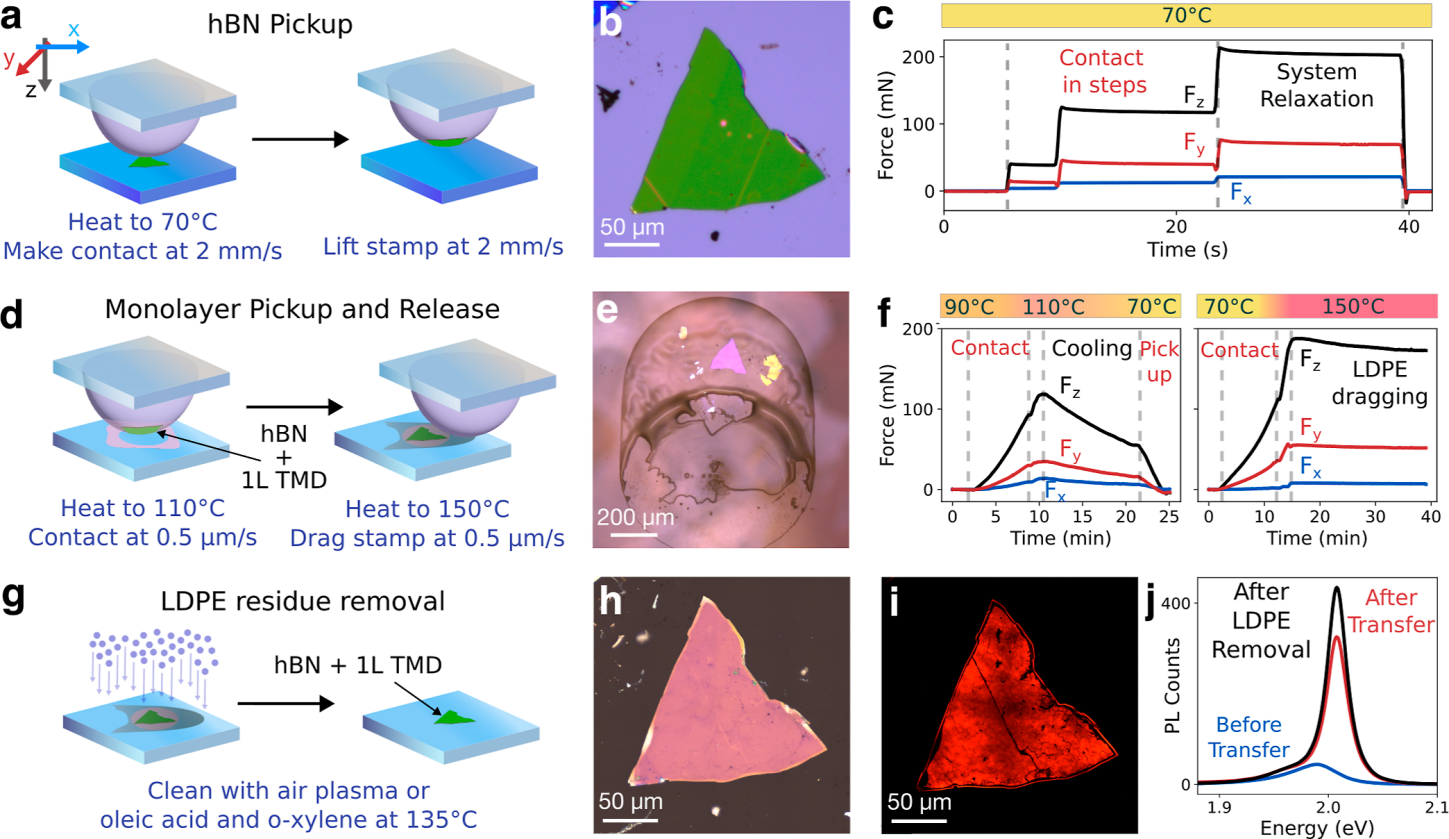
Transfer of hBN/monolayer heterostructures. (a) Procedure for hBN pickup. (b) Bright field image of the hBN flake before transfer. (c) Normal and in-plane force measurements during the pickup of an hBN flake. (d) Procedure for monolayer pickup with hBN and release on a target substrate. (e) Bright field microscope image of the flake after deposition, covered in LDPE residue. (f) Normal and in-plane force measurements during the pickup of the monolayer with hBN (left) and release of the structure on the final substrate (right). (g) Procedure for LDPE removal by air plasma treatment. (h) Bright field and wide field PL (i) images of the hBN flake after LDPE residue removal. (j) PL spectra before, after transfer, and after LDPE residue removal.

We pick up a mechanically exfoliated hBN flake from a Si substrate at 70 °C using an air plasma-activated LDPE stamp (100 W, 2 min), by making contact in steps of 0.1 mm at 2 mm/s (Figure 4a,b). While the step height remains constant, we observe an increase in the force exerted at each step, attributed to the larger contact area between the curved dome and the flat substrate (Figure 4c). After each step, we allow the system to relax for a few seconds, during which the force decreases slightly, visible as a small exponential relaxation following the initial force spike. When the hBN flake is fully covered by the LDPE, we lift the stage at a speed of 2 mm/s. We find that this method of making contact and picking up at large speeds induces fewer cracks in the hBN flake, as also observed in ref 33.

For the subsequent pickup of a WS_2_ monolayer with the stamp and hBN, we find that the relative humidity of the environment and the surface energy of the monolayer and carrier substrate play a significant role in the adhesion of the monolayer to the hBN flake. As such, we first cover the monolayer with a self-assembled monolayer (SAM) of 1-decanol (see Supporting Information Section 1 for further information), which passivates the surface and reduces the interaction with atmospheric contaminants,^43^ thereby enabling a clean monolayer pickup. The stamp with hBN is then put in contact with the monolayer at a temperature of 90 °C and a speed of 0.5 μm/s (Figure 4d), leading to an increase in *F*_*z*_ (Figure 4f, left). After contact, the structure is heated to 110 °C for 2 min, promoting the mobility of any air or water pockets trapped between the hBN and the monolayer and improving contact between layers. After cooling the stamp back to 70 °C, the stamp is pulled upward at a rate of 0.5 μm/s. In this process, both the 1L underneath and outside the hBN flake are in contact with the stamp is picked up. This presents a marked difference from the previously reported van der Waals pickup technique, where only the 1L in contact with hBN is picked up.^26^ Crucially, neither the top of the monolayer nor the bottom of the hBN flake has been in contact with the LDPE, which results in a clean heterostructure interface. To transfer the heterostructure to the target substrate, we employ a similar routine as for the single 1L transfer, with a contact at 70 °C at 0.5 μm/s followed by heating up to 150 °C to melt the LDPE. The stamp is then dragged at a speed of 0.5 μm/s and pulled up once the heterostructure is no longer in contact with the stamp, resulting in the sample covered by LDPE (Figure 4e). Due to the similarity of the procedures, the force curve of this transfer process (Figure 4f) closely mimics Figure 1c,f.

There are two different methods to remove the LDPE from the heterostructure. The first follows the procedure described above for monolayers, removing the LDPE with oleic acid and o-xylene at 135 °C. However, for structures stamped on substrates with minimal adhesion or large aspect-ratio features, potential solvent intercalation between the surface pattern and the heterostructure may result in detachment of the heterostructure from the surface. The second method, which we employ here, is to remove the LDPE with soft air plasma cleaning (Figure 4g). After this process, the hBN flake does not show visible damage (Figure 4h) and fully covers a continuous WS_2_ monolayer, as visible in the wide-field PL image (WFPL, Figure 4i). To assess any structural damage to the hBN flake after plasma etching, we perform AFM measurements that show no significant vertical etching of the hBN, only a subtle slanting of the side walls of the flake (Supporting Information, Section 6). We note that the difference in flake color between Figure 4b,h is due to a difference in the substrate (Si and SiO_2_ for b,h, respectively). To analyze the effect of the heterostructure transfer and subsequent plasma cleaning on the excitonic properties, we measure the PL of the structure (Figure 4j). After the transfer process, the line width decreases from 34 to 21 meV due to hBN passivation. Next, after the plasma cleaning step the exciton line width remains 21 meV, demonstrating that the underlying monolayer is not affected by the etching process. While the PL line width and trion contribution seem comparable before and after the removal of the LDPE, the overall intensity interestingly increases. We attribute this to the removal of the LDPE layer, whose rough surface caused reflection and scattering of light. Overall, we observe a 10-fold increase in the PL intensity as a result of the passivation and potentially a small Purcell enhancement due to internal reflections and interference patterns inside the hBN. We note that the decanol SAM present at the top interface of the monolayer may persist after heterostructure assembly, implying that the observed passivation could be due to the SAM rather than the hBN layer.^43^ We emphasize that the bottom interface of the monolayer is free of residues and SAM, and can therefore be used for heterostructure assembly with clean van der Waals interfaces. Similarly to the crack formation analysis performed for monolayer transfer, we find that 2% of the monolayer area is cracked before transfer, while after transfer we observe a small increase to 4%. Altogether, Figure 4 shows how the proposed transfer method also enables the direct transfer of large and high-quality hBN/monolayer heterostructures. We emphasize that the size of the heterostructure is only limited by the size of the hBN flake–which is mechanically exfoliated–and thus considerably smaller than the monolayer obtained through the GAE method.

To push the limits of the applicability of the presented method, we now explore the transfer onto high-aspect ratio structures. In particular, we transfer a 270 nm hBN/1L WS_2_ heterostructure onto a sapphire substrate patterned with 1.8 μm high domes (Figure 5). After transfer and plasma cleaning, we place the substrate in a vacuum oven at 300 °C for 1 h to promote the degradation of any residual LDPE trapped in between the sapphire domes.^44^

**Figure 5:**
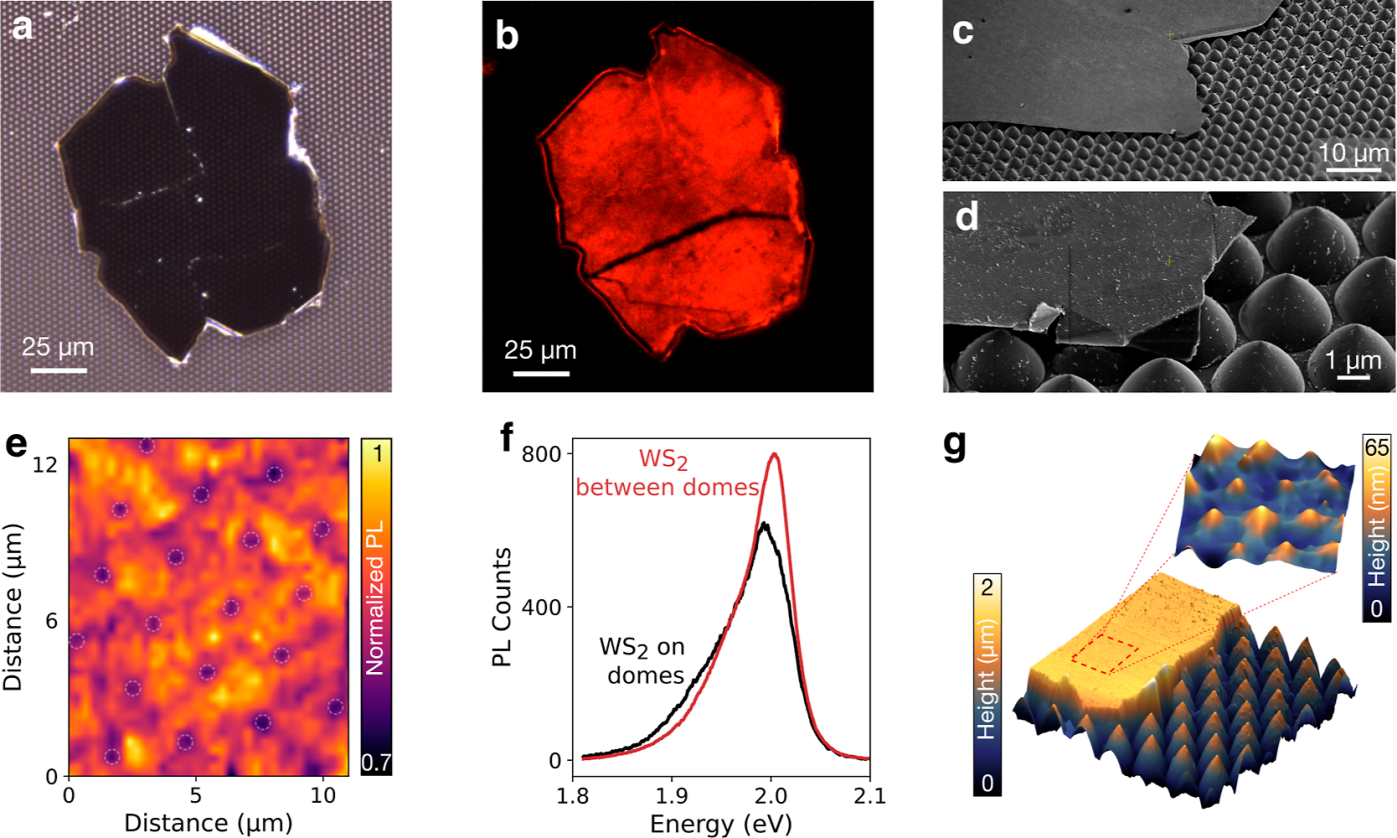
Transfer of a hBN/WS_2_ heterostructure onto a dome-patterned sapphire substrate. (a) Dark field and (b) WFPL microscope images of the heterostructure on the domes after the transfer and plasma cleaning. (c,d) Tilted SEM images of the heterostructure on top of the sapphire domes. (e) PL map of the heterostructure, where each pixel represents the integrated PL intensity. The points of contact are highlighted with white dashed circles. (f) PL spectra of the structure on tip and in between the domes. (g) AFM measurement of the heterostructure on top of the domes. Inset: small-area measurement of top of the flake.

Contact between the heterostructure and the substrate is limited to the tips of the domes, resulting in exceptionally small points of contact. To assess the effect of the transfer on the heterostructure, we record both dark field and WFPL images showing that although the WS_2_ monolayer is directly in contact with the domes, no signs of mechanical damage induced by the dragging process are observed (Figure 5a,b). The stripe where there is no WS_2_ under the hBN results from the exfoliation process, and was already present before transfer. Furthermore, SEM images of the heterostructure show that the flake lies suspended on top of the domes due to the high rigidity of hBN (Figure 5c,d).

To assess the impact of strain, we measure a PL map and spectra of the heterostructure on the domes (Figure 5e,f). The points of contact are clearly visible due to increased strain at these locations, causing a local 30% decrease of the PL emission. Comparing the PL spectrum of a bare WS_2_ monolayer on a flat substrate (Figure 2b, before transfer) with spectra on and between the domes, we see significant exciton broadening, a redshift and a larger trion contribution. These effects are in agreement with previous observations, where tensile strain applied to monolayer WS_2_ shows not only a redshift due to a reduced bandgap caused by lattice stretching,^45^ but also increased trion contributions.^46^ As expected, the PL spectrum on the domes shows a stronger redshift than in between domes, indicating that the tensile strain near the point contacts is larger. This interpretation is corroborated by an AFM scan of the structure (Figure 5g). While at larger scale the hBN flake appears to lie flat on the domes, the inset demonstrates that the flake drapes about 30 nm over the 3 μm separation between points of contact, inducing a strain of 3% at these regions. While the proposed transfer and cleaning method allows precise placement of large-area 2D material heterostructures on high aspect ratio structures, we also observe the presence of residue on the surface of the 2D material and the substrate. We emphasize that the solvent cleaning procedure outlined in Figure 1 should be employed in conventional transfer scenarios, while plasma cleaning should only be applied in patterns where solvent intercalation under the 2D material is possible, which could result in lifting and failure of the transfer. Overall, our results demonstrate that transfer of large-area hBN/monolayer heterostructures is possible even onto high aspect-ratio patterns, where the contact area between the materials and the substrate is extremely low, which is incompatible with most existing dry-transfer techniques.

### Applications of the Method

The two transfer techniques presented enable the assembly of high-quality, large-area 2D material structures and their combination with patterned or unpatterned substrates, which offers unique opportunities for techniques and device architectures which require large areas of continuous monolayer coverage. Here, we demonstrate two exciting possibilities of the proposed method (Figure 6): the fabrication of large-area bilayer heterostructures, and tunable optoelectronic devices with electronic control over the material’s charge carrier density.

**Figure 6:**
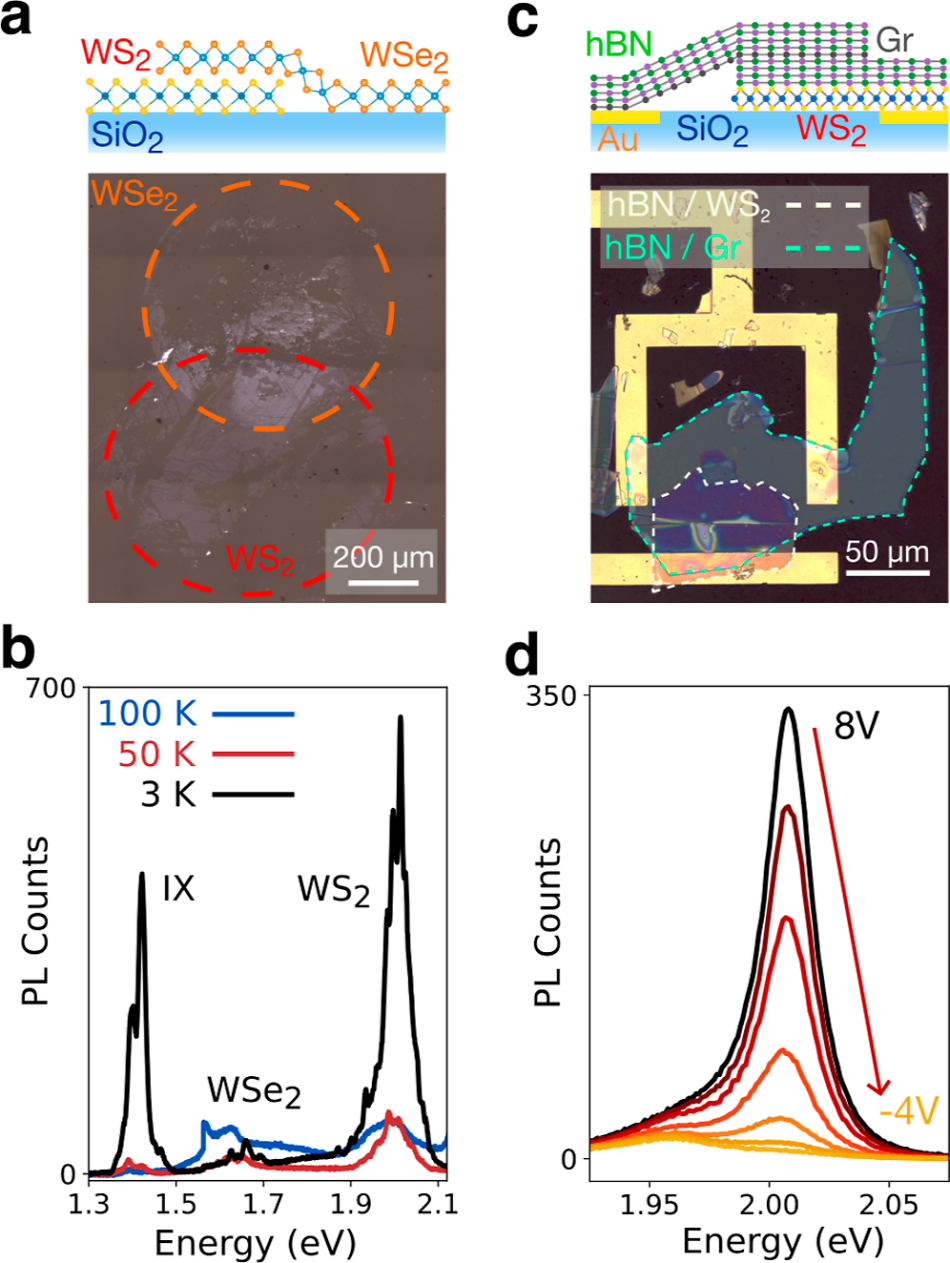
Demonstration of applications of the proposed transfer method. (a) Bright field microscope image of the WS_2_/WSe_2_ heterostructure. (b) PL spectra at temperatures of 100 (blue), 50 (red), and 3 K (black). (c) Bright field microscope image of the hBN/graphene/hBN/WS_2_ heterostructure stamped on Au contacts. (d) PL spectra at room temperature for different applied voltages.

First, we fabricate a large-area WS_2_–WSe_2_ heterostructure (Figure 6a). WS_2_ is transferred on a SiO_2_ substrate using the procedure outlined in Figure 1, followed by a similar transfer of WSe_2_. The heterostructure is then annealed in vacuum at 300 °C to promote contact between the materials. The two layers are stamped with partial overlap only, to provide areas for the characterization of the heterostructure as well as the bare WS_2_ and WSe_2_ monolayers as a reference. To probe the excitonic behavior of the heterostructure, we measure PL spectra at temperatures of 100, 50, and 3 K (Figure 6b). We observe the presence of the intralayer excitons of WS_2_ and WSe_2_ at 2 and 1.65 eV, respectively, and a third resonance at 1.4 eV (labeled IX), consistent with literature reports of interlayer excitons in WS_2_–WSe_2_ heterostructures.^47^ The three excitonic resonances demonstrate different behavior as a function of temperature. While both the WS_2_ intralayer exciton and the interlayer exciton increase their PL emission intensity at 3 K (as expected due to reduced exciton–phonon scattering), the WSe_2_ intralayer exciton is quenched. For the heterobilayer, the electron of the photogenerated excitons in WSe_2_ quickly transfers to the conduction band of WS_2_, while the hole remains in the valence band of WSe_2_. This spatial separation forms an interlayer exciton within a subpicosecond time scale, which exhibits a long lifetime.^47^ This mechanism directly suppresses the PL from WSe_2_ in the heterostructure as the electron transitions to the WS_2_ layer before it can recombine radiatively. The observation of interlayer exciton emission is a clear indication of electronic coupling between the layers, and thus that our method provides clean interfaces in the heterostructure assembly.

Next, we assemble a more complex heterostructure device including contacts and electrical gates to modify the charge carrier density of the material, enabling control of the optical properties of the heterostructure (Figure 6c). Using the procedure of Figure 4, fabrication of the device is achieved through a two-step process of transferring an hBN (100 nm)/WS_2_ heterostructure followed by an hBN (90 nm)/graphene heterostructure. The WS_2_ and the graphene connect to two different electrodes, and the middle hBN layer acts as an insulator, preventing short circuit below its breakdown voltage. The transfer of the hBN/graphene heterostructure highlights that the transfer method can also be applied to 2D monolayers other than TMDCs. Here, the high conductivity of the graphene enables its function as the gate electrode.^48^ We probe the effect of electrical gating on the exciton by measuring the PL at different gate voltages (Figure 6d). The PL of the WS_2_ monolayer can be fully quenched by inducing strong n-type doping at negative bias; we also observe PL enhancement as we approach 8 V. This increase in the neutral exciton emission with increasing positive bias indicates the possibility of intrinsic n-doping of the WS_2_ 1L, in agreement with previous results.^19^ These demonstrations show the broad range of possibilities enabled by the LDPE transfer method, both for fundamental studies of large-area 2D material heterostructures as well as the development of advanced optoelectronic devices.

## Discussion

We present a method for transferring 2D materials on patterned substrates, ranging from nanometer to micrometer sized gaps. While we demonstrate transfer of monolayer WS_2_ on 250 nm holes (Figure 3), there exists a gap size limit in which the applied strain during stamping is higher than the fracture strain, leading to monolayer rupture. To stamp in such large gaps, hBN is used for mechanical stability (Figure 5).

To obtain an overview of the possibilities enabled by the transfer method, we map the transfers by classifying them with contact fraction and strain, which are related to substrate adhesion and mechanical stress by the substrate’s pattern, respectively (Figure 7). From this analysis, we find that crack density is independent of contact fraction, consistent with LDPE being melted and transferred onto the substrate, making the process insensitive to 2D material–substrate adhesion. In contrast, strain strongly affects the crack percentage. To demonstrate the effect of strain beyond the reported fracture strain of 15–20%,^39^ we also show transfer of monolayers on the sapphire nanodomes and on a 600-grit ground glass diffuser. For monolayer on sapphire nanodomes, the dominant strain effect comes from contact strain with the tips of the domes, and these point contacts pierce the material. As such, PL is observed only from the base of the substrate, due to local WS_2_ rupture at the dome’s tip (Figure 7, top left corner inset). As previously shown, combining the monolayer with hBN allows transfer of a flat heterostructure on top of the domes, preventing piercing of the materials (Figure 5). A different scenario occurs for monolayer transfer onto a glass diffuser, where the large feature radius (21 μm) leads to low contact strain,^49^ but the spacing between grains (larger than 40 μm) imposes strain above the monolayer limit (Figure 7, middle inset). The monolayer locally conforms to each grain and ruptures in the regions between them, resulting in a crack percentage of 40%. Overall, Figure 7 illustrates how LDPE-based stamping enables the transfer of both monolayers and heterostructures across a wide range of topographies−highlighting where bare monolayers can support the induced strain and where mechanical support from thin hBN layers is essential. We note that optical metasurfaces operating in the visible/NIR spectral range typically exhibit feature sizes in the 10–300 nm range, well within the achievable range for our technique. Additionally, by judiciously choosing the hBN thickness, the resulting strain in the heterostructure can be engineered, which opens promising avenues for e.g. strain-induced single photon emitters.

**Figure 7:**
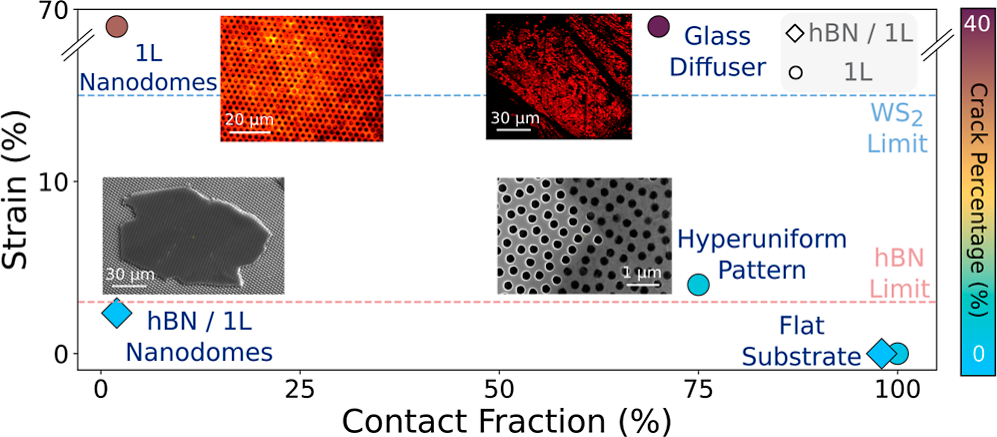
Strain and contact fraction map for multiple monolayer and hBN/monolayer transfers made with the LDPE-based transfer method. In the marker color, we encode the crack percentage induced by the transfer. We represent the literature reported fracture strain values for WS_2_ and hBN as horizontal dashed lines.

We present LDPE as a polymer carrier for 2D material transfer, for which polymers such as PDMS,^22^ PPC,^24^ and PVC,^23,33^ and others^50^ have also been used. Although these polymers have advantages for transferring specific 2D material layers, here we emphasize the particular attributes of LDPE that make it a suitable candidate for 2D material handling. Reliable 2D material transfer depends on precise tuning of the adhesion of the carrier polymer—high adhesion for pickup and low adhesion for release. Due to its 40–50% crystallinity, LDPE undergoes a melting transition upon heating as the crystalline domains collapse.^31^ In contrast, the previously studied amorphous polymers lack crystalline regions and therefore only soften above their glass transition temperature, before thermally degrading.^51^ The thermal melting drives the LDPE into a low-adhesion state, essential for successful transfer. The large difference in adhesion regimes offered by the LDPE phase transition is responsible for the exceptionally high success rate obtained with this method.

A useful comparison for our LDPE-based method is provided by standard wet-transfer techniques for 2D materials. In typical wet transfer, cm-scale monolayers are released by dissolving a polymer support in a liquid and collecting the floating film from the liquid–air interface.^52^ While GAE monolayers are usually limited by cracks in the original crystal (Figure 1b), they still yield nearly mm-sized, continuous flakes that are suitable for large-area optoelectronic devices.^28^ Despite the smaller area, our approach offers two key advantages over wet transfers. First, it enables deterministic placement of 2D materials on patterned substrates or on top of other 2D layers, which is highly challenging with conventional wet-transfer alignment. Second, many wet-transfer protocols result in a strong trion contribution in the TMDC photoluminescence,^53,54^ likely due to doping induced by the liquid medium. Our LDPE-transfer method allows monolayer placement with minimal trion emission, allowing the transfer of high-quality 2D materials onto patterned substrates.

We observe that the process of monolayer pickup using hBN, outlined in ref 26 is highly dependent on the environmental relative humidity (RH), with both low (below 20%) and high (around 60%) RH values resulting in unreliable pickup. Here, the low surface energy of the bare LDPE, along with the possibility to increase it through plasma activation,^32^ provides a strong advantage by enabling careful tuning of the adhesion to match the necessary requirements. We verify that the plasma activation step along with covering monolayers with a 1-decanol SAM provides a robust protection against RH variations, making the pickup of hBN/monolayer structures a reliable and crack-free process, essential for optoelectronic devices.

While we focused on the transfer of GAE monolayers due to their intrinsically high quality and large area, we also highlight the limitations of 2D materials exfoliated using this method. As previously mentioned, the area that can be effectively used for optoelectronic devices is limited by pre-existing cracks in the original bulk crystal, which in practice reduces reduces the continuous monolayer size to between hundreds of μm and the mm scale (Figure 1b).^28^ Furthermore, not all 2D materials can be exfoliated using this technique. Notable examples of widely used 2D materials that cannot be easily exfoliated via GAE are graphene and hBN. We verify that the proposed transfer technique also works on large-area CVD-grown materials, showing similar increase in PL emission as seen in Figure 2b (see Supporting Information Section 7).

Finally, we emphasize that while the force sensors employed in our transfer stage offer quantitative insights in the forces at play during the stamping process, the fundamental process does not rely on these sensors. As such, a simple motorized translation stage combined with an inexpensive temperature controller for the sample holder will readily enable the high-yield transfer of large-area monolayers and heterostructures.

## Conclusions

In summary, we present a simple and versatile transfer method that capitalizes on the temperature-dependent adhesion and viscosity of LDPE to enable the transfer of large-area 2D material flakes, including GAE monolayer 2D materials and their heterostructures with hBN, onto substrates with surface textures. We show the transfer of both large monolayers as well as hBN/monolayer heterostructures onto patterned metasurfaces and sapphire domes to illustrate that even the most extreme surface textures that offer minimal adhesion can serve as the target substrate. This is in stark contrast to traditional methods which strongly rely on a flat surface with sufficient adhesion. We demonstrate the application of the proposed method in combination with several patterned substrates to enable novel optical functionalities, including tailored angular emission patterns as well as strain- and electrostatic modulation of over excitonic emission. By fabricating large-area heterobilayers, we additionally confirm electronic coupling of the layers through the observation of interlayer exciton emission. We envision that this transfer method opens avenues for the integration of large area (monolayer) 2D materials with nanostructured surfaces, enabling the exploration of new 2D materials physics and the development of novel optoelectronic devices that leverage the unique properties of atomically thin 2D semiconductors.

## Methods

### Exfoliation of 2D Materials

Using the Au assisted exfoliation method reported previously,^27^ we obtain large-area monolayers for transfer. A 100 nm Au layer is deposited on a cleaned Si wafer at a rate of 0.5 Å/s, using electron beam physical vapor deposition (Polyteknik Flextura M508 E). A 300 nm layer of PMMA is then spin coated on top of the Au to provide more mechanical stability during the peeling process. We use thermal release tape (TRT, Revalpha RAY-4LSC­(N), Nitto Denko Corporation) and pick up the PMMA/Au layers, after which we press it immediately against a bulk TMDC crystal (WS_2_ and WSe_2_, HQ Graphene). After the exfoliation, we transfer the monolayer to cleaned SiO_2_ substrates. The substrates are then heated to 110 °C to remove the TRT, cleaned with acetone to remove the PMMA and placed in Au etchant (651818, Sigma-Aldrich) for 2 min to remove the gold. Finally, the sample is washed in isopropanol and dried with a nitrogen gun.

hBN flakes are manually exfoliated from the bulk crystal (HQ Graphene) using Nitto SPV-224 tape and transferred to a Si substrate using TRT.

For the gated device, commercial large-area graphene monolayer is used (CVSO1011, ACS Material).

For fabrication of hBN/monolayer heterostructures, the large-area monolayers are covered in 1-decanol following the procedure outlined in ref 43 The substrates with monolayers are immersed in 1-decanol (8034631000, Sigma-Aldrich) for 2 min at 160 °C. After this, the substrates are rinsed in IPA and dried using N_2_.

### Fabrication of the Stamps

The stamp is fabricated starting with a glass slide covered with two layers of PDMS with different thicknesses, cut from Gel Pak WF-40 × 40-0060-X0-A and AD-22T-00-X4. On top of the PDMS, a drop of heat-resistant superglue is placed and left to dry overnight. A square of LDPE film (commercially available as kitchen cling film, “G’woon vershoudfolie”) is then cut from the cling film and stretched over the stamp, so that it conforms to the superglue drop. The LDPE film is held onto the stamp using Scotch tape. Details and illustrations on the stamp fabrication are provided in Section 1 of the Supporting Information. For fabrication of hBN/monolayer heterostructures, the fabricated stamps are placed in an air plasma chamber at 100 W for 2 min before use. The same plasma settings are used for LDPE cleaning after stamping.

### Stamping Setup and Force Measurements

For stamping of the 2D material flakes, a custom-built stamping microscope is developed. The substrate with GAE monolayer is placed on a heater that allows temperature control. The stamp is manipulated by a *xyz* motorized platform (MT1-Z9, Thorlabs), allowing full control over direction and velocity of the motion. The process can be imaged through the stamp using either a Nikon Plan Fluor 4× (NA = 0.13) or 10× (NA = 0.3) objective.

The stamping tool is equipped with six load cells (SparkFun 14727) incorporating a Wheatstone bridge configuration to detect changes induced by mechanical strain. The signal is read using an HX711 load cell amplifier, performing analog-to-digital conversion. The amplifier is then interfaced with a microcontroller for real-time force monitoring and control.

### Spectroscopic Measurements

Room temperature spectroscopic measurements are performed using a Witec α300R confocal Raman microscope. Here, a Zeiss EC Epiplan Neofluar 100× BD (NA = 0.9) is used with a 532 nm laser as the light source. For the BFP measurements, a Zeiss Plan-Apochromat 100× (NA = 1.4) oil-immersion objective is used to image beyond the light line in air.

For the wide field PL images a 405 nm laser focused on the back focal plane of the objective is used, providing wide field near-normal illumination of the sample. The laser light is then filtered by a 600 nm long-pass filter, allowing real space imaging of the sample’s PL.

Measurements at cryogenic temperatures are performed using a custom-built optical setup and a Montana Cryoadvance 50 cryostat, using an in-vacuum Zeiss EC Epiplan-Neofluar 0.9NA 100× objective for PL measurements.

### Electrical Connections and Gating Measurements

To enable electrical contacts during optical characterization, the sample is mounted onto a custom printed-circuit board (PCB) using double-sided tape. The gate and ground electrodes are wire-bonded to separate contact pads on the PCB using 25 μm diameter aluminum wires (West Bond 7KE). For DC gating measurements, a gate bias is applied using a Keithley 2612B SourceMeter. The experiment is fully automated using custom Python scripts to control the equipment. Initially, the sample is tested to establish the limits of maximum PL enhancement (+4 V) and full exciton quenching (−8 V). The voltage was subsequently swept from +4 to −8 V in 1 V increments, registering a maximum leakage current of 0.1 nA. We observe a hysteresis effect, especially noticeable at measurements at 0 V, in agreement with previously reported work.^19^

### Device Characterization

For the AFM measurements, a Bruker Dimension FastScan system is used at a rate of 5 μm/s. The SEM images are taken in a Thermo Fisher Scientific Helios UX 5 system. For the hBN/WS_2_ on sapphire domes, the sample is first coated with a 5 nm Au layer using a sputtering tool to prevent charge accumulation.

### Patterned Surfaces

The hyperuniform pattern is fabricated on a cleaned Si substrate using electron beam lithography (Raith Voyager lithography system) to pattern the nanoholes in a CSAR resist layer. After development, the nanoholes are etched in the Si using Cl_2_ + HBr/O_2_ plasma at a rate of 3.3 nm/s (Oxford PlasmaPro100 Cobra). Lastly, a SiO_2_ layer is grown on the sample using rapid thermal annealing (AnnealSys AS-one 100) at 1100 °C under O_2_ atmosphere (50 sccm). The substrate with sapphire domes is purchased from MSE Supplies (WA0433). A 600-grit BK7 ground glass diffuser was also used (Thorlabs DG10–600).

## Supplementary Material



## Data Availability

A full replication package, including all data and analysis scripts, is freely available on 10.6084/m9.figshare.32857916.

## Notes

The Supporting Information is available free of charge online and contains descriptions and details of: fabrication of stamps for 2D material transfer, the stamping tool and force measurements, spectral analysis of the PL and Raman spectra at multiple transfer steps, strain calculation from AFM data, calculation of the structure factor and directional gain for the hyperuniform metasurface, AFM measurement of hBN flake thickness before and after plasma etching, transfer of CVD grown monolayers (PDF)

## Notes

Notes: A preprint version of this article was deposited at arXiv.org: Dias, B. S.; Dziobek-Garrett, R.; Mentasti, G.; Gupta, A.; Lambertz, A.; Alarcón-Lladó, E.; Schall, P.; Bliem, R.; van de Groep, J. “Large area deterministic stamping of 2D materials on arbitrarily patterned surfaces” arXiv (Mesoscale and Nanoscale Physics), March 4, 2026, 2603.04623, ver. 1.https://arxiv.org/abs/2603.04623 (accessed July first, 2026).
