# Supplementary material for: Large-Area Deterministic Stamping of 2D Materials on Patterned Surfaces
Source: ACS Nano. 2026 Jul 12;20(29):20611–22. doi: 10.1021/acsnano.6c04231 (PMC13421966; doi:10.1021/acsnano.6c04231)
Supplement: Supplementary file 1 [file nn6c04231_si_001.pdf]

Supplementary Information for: Large-Area Deterministic Stamping of 2D Materials on Patterned Surfaces

Bernardo S. Dias,[†] Reynolds Dziobek-Garrett,[‡,†] Gabriella Mentasti,[†] Abhishek Gupta,[†] Alexander Lambertz,[¶,†] Esther Alarcón-Lladó,[§,¶] Peter Schall,[†] Roland Bliem,[‡,†] and Jorik van de Groep[*,†]

†*Van der Waals-Zeeman Institute, Institute of Physics, University of Amsterdam, Amsterdam, 1098 XH, the Netherlands*

‡*Advanced Research Center for Nanolithography, Science Park 106, 1098 XG Amsterdam, the Netherlands*

¶*Center for Nanophotonics, AMOLF, 1098 XG Amsterdam, The Netherlands*

§*van 't Hoff Institute for Molecular Sciences, Universiteit van Amsterdam, Amsterdam, 1098 XH, the Netherlands*

E-mail: j.vandegroep@uva.nl

1 LDPE Stamp Fabrication

Here we detail the fabrication of the stamps used for the transfer of monolayer and hBN / monolayer heterostructures. The LDPE is stretched and placed on a glass slide, and cut in square shape with a sharp knife, (Fig. S1a-b). A glass slide is then prepared with a double PDMS layer, with the bottom layer cut from Gel Pak WF-40×40-0060-X0-A and the top one from AD-22T-00-X4 (Fig. S1c). These layers ensure good adhesion of the whole structure to

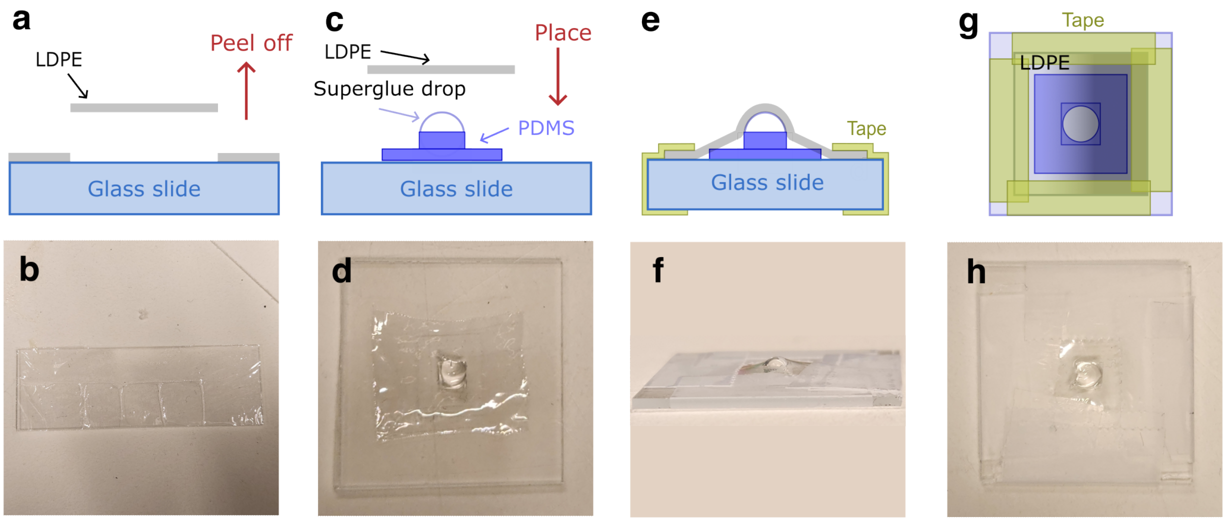

Figure S1: Fabrication of the stamps for 2D material transfer. **(a,c,e,f)** Schematics of the different steps of stamp fabrication. **(b,d,f,h)** Photographs of the stamp at each step of the fabrication.

the glass and create an offset distance to prevent the stamp from touching the 2D materials anywhere else except from the dome top. A drop of superglue is then manually placed on top of the thicker PDMS and left overnight to dry. The PDMS-superglue stack also creates a soft set of polymers that allows the stamp to conform to the 2D material without applying too much pressure, which could lead to cracks in the transfer. The LDPE layer is then placed on the structure (Fig. S1d) and streched with tape, ensuring good contact between the LDPE and superglue (Fig. S1e-f). Figures S1g and h demonstrate the final result from stamp fabrication.

As mentioned in the main text, for the hBN / 1L pickup we perform an air plasma activation of the LDPE after stamp fabrication and additionally create a decanol self assembled monolayer on the 2D material monolayer, as described in the main text. Without this step, we verify that the pickup of monolayers under hBN is not successful, possibly due to surface energy mismatch between the materials and the stamp, leading to trapping of interfacial water or contaminants. Figures S2a and b show an attempt to pick up graphene with hBN. We verify that after the procedure described in the main text, a large portion of the graphene was picked up (where it was contacting with LDPE), exposing the SiO_2/Si

substrate. Nevertheless, in the regions where the hBN flakes were present, we see a perfect outline of the flake shape, indicating that the graphene remained in the substrate and that the pickup failed.

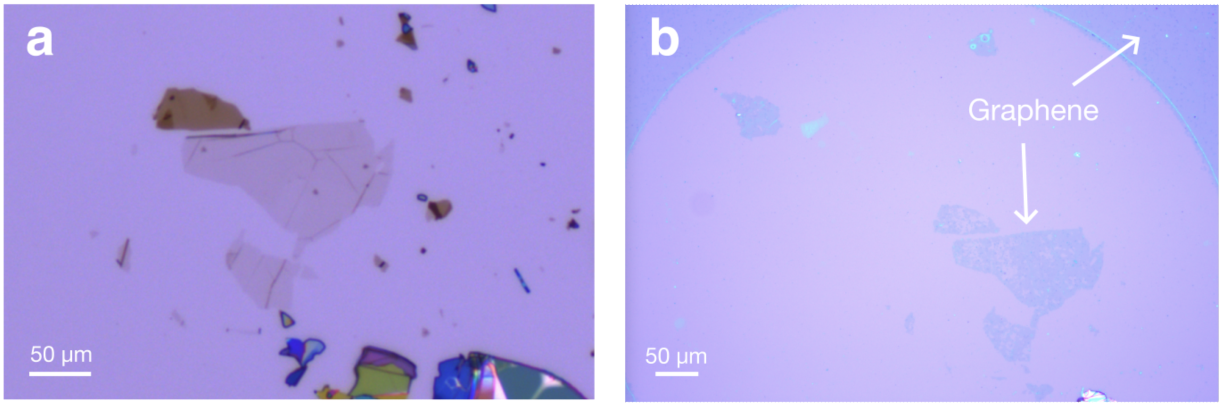

Figure S2: Attempt to pick up monolayer graphene with hBN without plasma and decanol treatment. **(a)** hBN flakes used in the attempt. **(b)** Graphene layer after pickup attempt, where the shape of the hBN flakes is clearly visible.

2 Stamping tool and force measurements

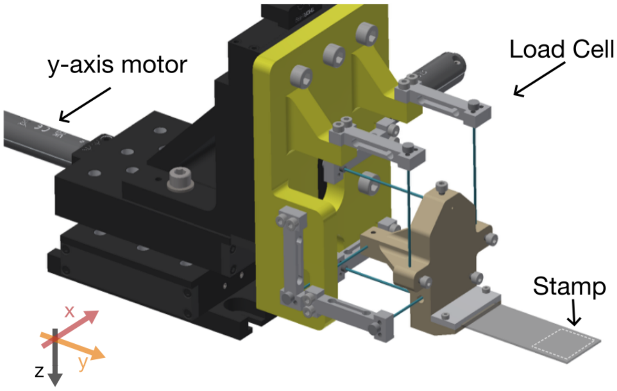

Figure S3: Schematic of the stamping tool equipped with load cells for force measurement of 2D material transfer.

Stamping is performed using a motorized stamping tool in x, y and z directions, allowing precise control of the contact with the monolayer (Fig. S3). The stamping tool includes

six load cells that hold the stamp and allow for independent measurement of the forces along the three axes. The stamp is suspended along the y axis, allowing contact with the monolayer without interference from the rest of the tool. It should be noted that under this geometry, for forces that induce some curvature on the glass slide, F_y and F_z are not fully independent. This coupling is caused by the deformation of the glass slide under because the beam's deflected shape increases its arc length, requiring an axial support force to satisfy the glass inextensibility, so a change in F_z inherently leads to a generation of a force in the y direction (axial).[1]

3 Spectral analysis of the transferred monolayer

To explain the change in the PL spectra between the different transfer steps, we turn to a deeper analysis of the spectral lineshape and peak positions (Fig. S4).

From Fig. 2b of the manuscript, it is apparent that the largest change occurs between the as-exfoliated sample on a SiO_2 substrate and its subsequent pickup and transfer to a new SiO_2 substrate, still with LDPE residue on top. On that path, the radiative rate of neutral excitons, assigned to the intense peak around 2.02-2.03 eV emerges and vastly exceeds that of the trion peak around 1.98-2.0 eV. Such a change is characteristic of a large change in the strain or Fermi level in the monolayer.

First, we address possible contributions from strain by fitting Lorentzian contributions at the known WS_2 peak positions to the Raman spectra at different steps of the transfer. Comparing the Raman spectra of the WS_2 monolayer on the gold pickup to the same monolayer on a SiO_2 substrate after the GAE is complete, we see a hardening by 2.2 cm^{-1} of the E' peak around 354 cm^{-1}, which is an in-plane vibration and most sensitive to strain (Fig. S4a,b).[2,3] This mode remains stable upon stamping and transfer with LDPE to a new SiO_2 substrate. On the other hand, the A'_1 mode (416 cm^{-1}) shifts to higher wavenumbers after stamping, indicating a reduction in the doping level of the monolayer.[4] We therefore argue

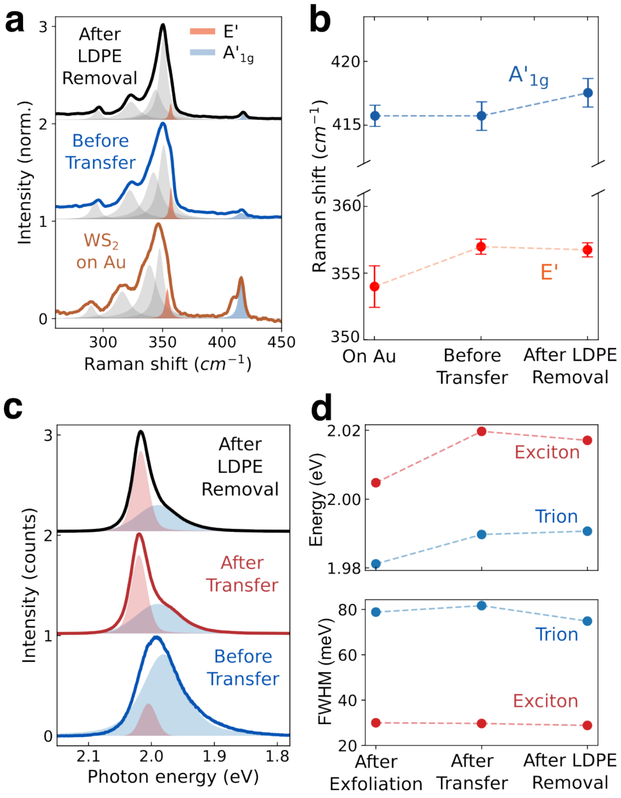

Figure S4: Analysis of the monolayer WS_2 Raman and PL spectra in the different steps of transfer. **(a)** Raman spectra with the respective Lorentzian line fits. **(b)** Raman shift of the A'_{1g} and E' peaks at different steps of the transfer. **(c)** PL spectra with the respective Lorentzian line fits. **(d)** Neutral exciton and trion peak energies and FWHM at different steps of the transfer.

that a change in the strain is unlikely to be the origin of the shift from trion-dominated to neutral exciton-dominated PL upon transfer.

The changes to the fitted peak positions of the neutral exciton and especially trion contributions also point to doping as the largest factor in changing the PL signature (Fig. S4c,d). Specifically the continued blue shift in the trion peak throughout the process is indicative of a continued change in the Fermi level.[5]

There are further competing mechanisms when assigning the origin of the shift in doping level of the monolayer through the stamping process. We rule out contributions from the substrate, as both the initial and targets are SiO_2, cleaned in the same way and not exposed to high temperatures and aggressive environments which are known to alter its

surface chemistry. This leaves solvents from the removal of the PMMA and Au from the initial exfoliation and passivation of defects and charge neutralization by oleic acid, which we use to remove the LDPE stamp. Because the largest change comes upon removal from the initial substrate, we argue that the intercalated polar solvents are likely the cause of the initial doping and thus trion-dominated PL. Poddar et al. have shown that exposure to acetone and IPA cause unintentional doping seen by large shifts to the threshold voltage of monolayer Mo_2 transistors.[6]

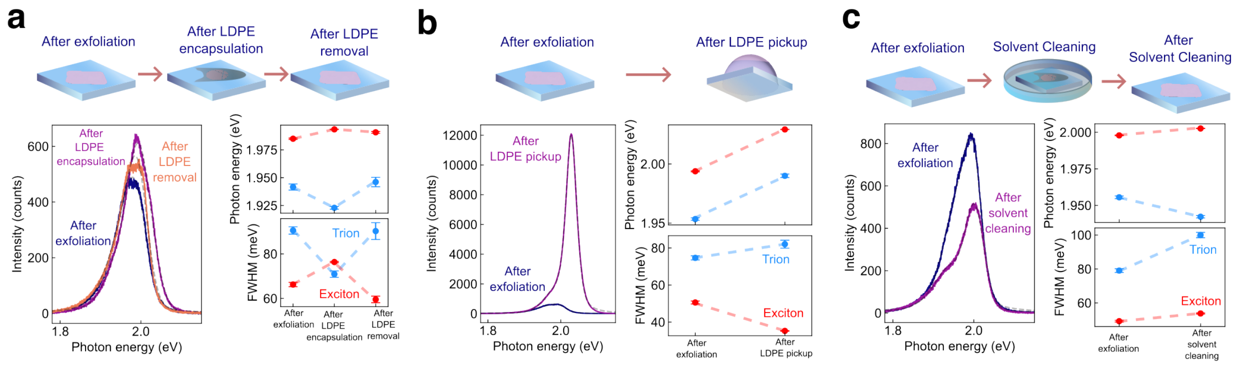

Figure S5: Analysis of the monolayer WS_2 PL spectra under different encapsulation and chemical treatments. (a) PL spectra before, after LDPE encapsulation and after LDPE cleaning with oleic acid and o-xylene. **(b)** PL spectra before and after pickup with the LDPE stamp (measured on the stamp). **(c)** PL spectra before and after solvent cleaning (no pickup).

Finally, we decouple the possible effects of charge transfer from the LDPE stamp. As WS_2 monolayers are known to be slightly n-doped due to the presence of chalcogen vacancies, the interaction between the LDPE stamp and the monolayer may also induce a reduction in the Fermi level through charge transfer.[7–9] This we also cannot decouple from the affects of the oleic acid, with which we remove the LDPE stamp, which is also known to passivate chalcogen vacancies and neutralize MoS_2 and WS_2. We conduct three control experiments to understand the roles of removal from the initial substrate, possible charge transfer from LDPE, and passivation from oleic acid (Fig. 5). We measure PL from three samples, first from a WS_2 monolayer on the LDPE stamp just after pickup, second from a WS_2 encapsulated in LDPE but not picked up or transferred, and third from a monolayer which

was rinsed with oleic acid and O-xylene.

We observe the largest exciton blue-shift and intensity enhancement for the monolayer just after liftoff from its parent substrate, as measured still on the LDPE stamp (Fig. 5b). In contrast, neither encapsulation in LDPE or washing with OA or O-xylene causes any significant enhancement in PL intensity (although solvent cleaning reduces the trionic contribution). Raman spectra of the WS_2 monolayer before and after pickup from the parent substrate point to a change in the free carrier density as the biggest contributor to the changes in PL we observe. While the E' mode hardens by just 0.25 ± 0.15 cm^{-1}, the A_1' mode hardens by 2.10 ± 0.83 cm^{-1}, which is consistent with previous studies showing stronger dependence of the A_1' on carrier doping than the E' mode.[4]

4 Modelling strain from AFM data

In the transfer of 2D materials there are two main causes of strain in the transfer. First, when a 2D material is transferred over a gap of length L, this unsupported span will cause a deflection and will make the flake behave like a strained membrane of length s, causing loading strain ε_L on the material. Secondly, the points of contact might be sharp and possess small radius of curvature, causing localized strain on the 2D material and possibly piercing through, as seen in Fig. 7, top left inset. We refer to this effect as contact strain ε_c. In a 2D material transfer, we model these two effects as different sources of strain and consider a successful transfer if the strain on the 2D material $\varepsilon = max(\varepsilon_L, \varepsilon_c) < \varepsilon_f$, where ε_f is the fracture strain of the 2D material, between 10 to 15% for monolayers[10] and 3% for hBN.[11] This model implies that if the applied strain (load or contact) is smaller than the fracture strain, the transfer should be successful.

Defining strain as $\varepsilon = \Delta L/L = (s - L)/L$, we use AFM data for both monolayer transfer and hBN / monolayer transfer to extract the arc length s by fitting a parabola $z = ax^2$ to the data over the unsupported gap, as seen in the inset of Fig. 3f. Calculating s as the arc

length

$$s = \int_{-L/2}^{L/2} \sqrt{1 + \left(\frac{dz}{dx}\right)^2} dx \approx \int_{-L/2}^{L/2} 1 + \frac{1}{2}\left(\frac{dz}{dx}\right)^2 dx$$

we obtain $\varepsilon_L = a^2L^2/6$. We note that this calculation is fully consistent with the Föppl–von Kármán formalism, where $\varepsilon_{xx} = \frac{du_x}{dx} + \frac{1}{2}\left(\frac{dz}{dx}\right)^2$ and u_x is the in-plane displacement.[12] Since the strain applied is purely geometric, we ignore the in-plane contribution, obtaining $\varepsilon_{ii} \approx \frac{1}{2}\left(\frac{dz}{dx}\right)^2$. Thus, the strain calculation via the parabolic approximation corresponds to the average strain along the suspended 2D material.

5 Calculation of the structure factor

In the hyperuniform pattern presented in the main text, the structure factor is calculated using images obtain from a SEM.

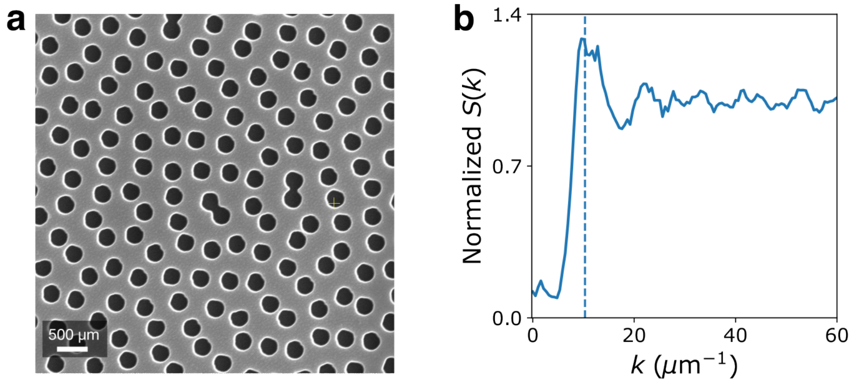

Figure S6: Calculation of the structure factor S(k) of a hyperuniform pattern. (a) SEM image used for calculation of S(k). **(b)** Calculated S(k). The dashed line corresponds to the maximum value registered in the BFP PL image of the device.

Taking Fig. S6a, the structure factor S(k) was calculated using Fiji[13] to threshold and extract the position of the center of each nanohole. To calculate S(k), we used[14]

$$S(\mathbf{k}) = \frac{1}{N}\left\langle \sum_{k,j=1}^{N} e^{-i\mathbf{k}\cdot(\mathbf{R}_j - \mathbf{R}_k)} \right\rangle,$$

where $\mathbf{k}$ is the wavevector, N is the total number of nanoholes and $\mathbf{R}_{j,k}$ are the positions

of each nanohole. The maximum of $S(\mathbf{k})$ is obtained at $k = 10.3\ \mu\text{m}^{-1}$, in agreement with the BFP measurements shown in the main text, that demonstrate PL enhancement at this value of the wavevector (Fig. S6b).

For the normalization of the angularly resolved PL emission via BFP measurements, we calculate first calculate the power normalized the angular average intensity $\tilde{I}(k)$:[15]

$$\tilde{I}(k) = \frac{I_\theta(k)}{\int_0^{2\pi} \int_0^{k_{max}} I_\theta(\theta, k) dk d\theta}$$

where $I_\theta(k)$ is the acquired BFP image. We now perform normalization with a substrate of the same materials without the pattern, obtaining the directional gain γ_{dn}:

$$\gamma_{dn} = \frac{\tilde{I}(k)}{\tilde{I}(k, ref)}$$

6 Plasma removal of LDPE residue

Using AFM measurements, we demonstrate that the air plasma approach does not induce any thickness change in the hBN flakes, allowing this method to be used in nanophotonic applications, where dielectric thickness can be crucial (Fig. S6).

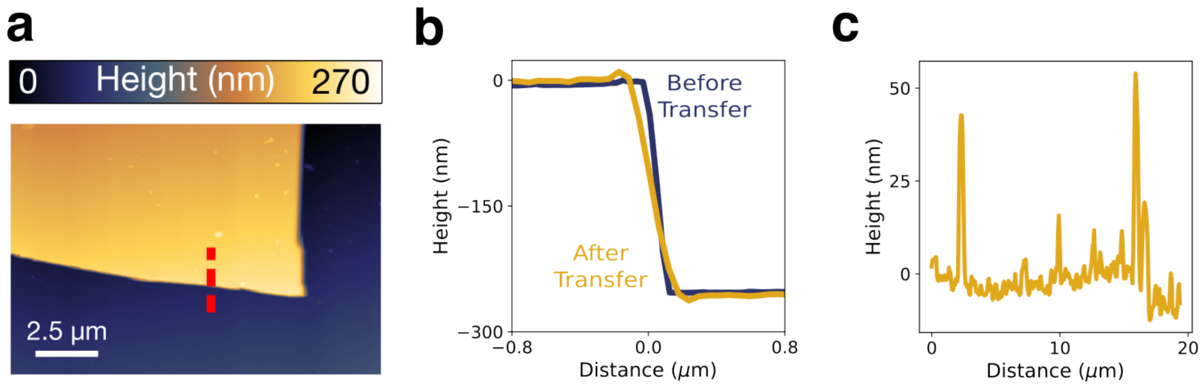

Figure S7: AFM scan of the hBN flake used for monolayer transfer. (a) Full AFM scan of the hBN flake before transfer. The red dashed line indicates the cross section used in (b). **(b)** Thickness measurement before and after air plasma removal of LDPE. **(c)** Height profile of the hBN surface after plasma cleaning.

We verify that no significant vertical etching of the hBN flake is observed, only a slight tilt of the hBN wall can be obtained for prolonged plasma exposure, this effect is not significant for most applications because the hBN lateral size is much larger than the scale of this effect. Analyzing the hBN surface data, we observe that large scale particulates (around 50 nm in height) are present at very low densities. Based on this data, we calculate a standard deviation of the surface height of 2.2 nm, showing that the residue present is mostly of small scale.

7 Transfer of CVD-grown monolayers

In the manuscript we focused on transfer of GAE monolayers due to their inherent high-quality and large areas, enabling combination with large patterned surfaces. While applicable in many scenarios, not all materials can be exfoliated using this technique, and CVD growth remains one of the most common methods to obtain large area monolayers. Here, we demonstrate that the same monolayer transfer method used in the manuscript applies also for CVD grown materials.

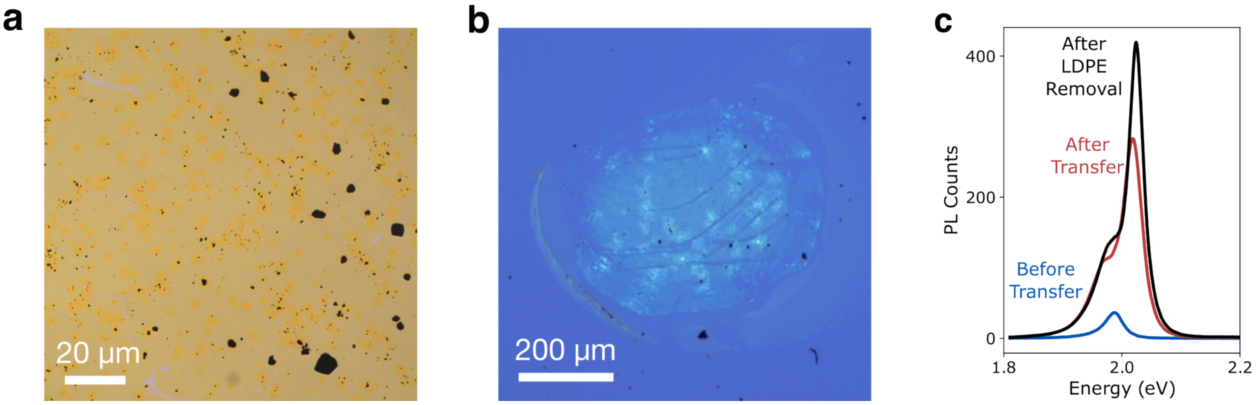

Figure S8: Transfer of CVD-grown WS_2 monolayer. (a) Bright field image of the CVD-grown WS_2 monolayer, before pick-up. The darker yellow patches correspond to bilayer growth, while the dark spots correspond to the seed crystals used for monolayer growth. **(b)** Bright field image of the transferred WS_2 monolayer. **(c)** PL spectra of the monolayer before and after transfer, as well as after LDPE residue removal.

CVD-grown WS_2 on SiO_2 (300 nm) / Si was transferred to a substrate of the same

materials, following the recipe discussed in the manuscript. Overall, we observe similar results to the GAE transfer, with full monolayer and bilayer pickup and transfer to the target substrate (Fig. S7a,b). Analysis of the PL (Fig. S7c) demonstrates a similar trend after transfer, with a 12x increase of the PL intensity followed by a linewidth reduction from 40 meV (before transfer) to 27 meV (after LDPE removal). Here, a marked difference to the GAE-exfoliated monolayer after transfer is the much stronger trionic contribution, indicative of possible defects caused by the growth process of the CVD monolayer.

References

(1) Beléndez, T.; Neipp, C.; Beléndez, A. Large and Small Deflections of a Cantilever Beam. *Eur. J. Phys.* **2002**, *23*, 371.

(2) Michail, A.; Anestopoulos, D.; Delikoukos, N.; Grammatikopoulos, S.; Tsirkas, S. A.; Lathiotakis, N. N.; Frank, O.; Filintoglou, K.; Parthenios, J.; Papagelis, K. Tuning the Photoluminescence and Raman Response of Single-Layer WS_2 Crystals Using Biaxial Strain. *J. Phys. Chem. C* **2023**, *127*, 3506–3515.

(3) Negi, D.; Baishya, M.; Moghe, A. R.; Paul, S.; Badola, S.; Saha, S. Uniaxial Strain-Dependent Resonant Raman Scattering in WS_2. *Small* **2025**, *21*, 2412832.

(4) Sohier, T.; Ponomarev, E.; Gibertini, M.; Berger, H.; Marzari, N.; Ubrig, N.; Morpurgo, A. F. Enhanced Electron-Phonon Interaction in Multivalley Materials. *Phys. Rev. X* **2019**, *9*, 031019.

(5) Mak, K. F.; He, K.; Lee, C.; Lee, G. H.; Hone, J.; Heinz, T. F.; Shan, J. Tightly Bound Trions in Monolayer MoS_2. *Nat. Mat.* **2013**, *12*, 207–211.

(6) Poddar, P. K.; Zhong, Y.; Mannix, A. J.; Mujid, F.; Yu, J.; Liang, C.; Kang, J.-H.; Lee, M.; Xie, S.; Park, J. Resist-Free Lithography for Monolayer Transition Metal Dichalcogenides. *Nano Lett.* **2022**, *22*, 726–732.

(7) Shokouh, S. H. H.; Jeon, P. J.; Pezeshki, A.; Choi, K.; Lee, H. S.; Kim, J. S.; Park, E. Y.; Im, S. High-Performance, Air-Stable, Top-Gate, p-Channel WSe_2 Field-Effect Transistor with Fluoropolymer Buffer Layer. *Adv. Funct. Mater.* **2015**, *25*, 7208–7214.

(8) Ma, J.; Choi, K.-Y.; Kim, S. H.; Lee, H.; Yoo, G. All Polymer Encapsulated, Highly-Sensitive MoS_2 Phototransistors on Flexible PAR Substrate. *Appl. Phys. Lett.* **2018**, *113*, 013102.

(9) McGinn, C. K.; Harrington, D. M.; Heilweil, E.; Hacker, C. A. Spectroscopic Analysis of Polymer and Monolayer MoS_2 Interfaces for Photodetection Applications. *Appl. Phys. Lett.* **2024**, *124*, 012106.

(10) Falin, A.; Holwill, M.; Lv, H.; Gan, W.; Cheng, J.; Zhang, R.; Qian, D.; Barnett, M. R.; Santos, E. J. G.; Novoselov, K. S.; Tao, T.; Wu, X.; Li, L. H. Mechanical Properties of Atomically Thin Tungsten Dichalcogenides: WS_2, WSe2, and WTe_2. *ACS Nano* **2021**, *15*, 2600–2610.

(11) Zhou, J.; Zhu, M.; Han, Y.; Zhou, X.; Wang, S.; Chen, J.; Wu, H.; Hou, Y.; Lu, Y. Direct Measurement of Tensile Mechanical Properties of Few-Layer Hexagonal Boron Nitride (h-BN). *J. Appl. Phys.* **2024**, *135*, 224301.

(12) Darlington, T. P.; Krayev, A.; Venkatesh, V.; Saxena, R.; Kysar, J. W.; Borys, N. J.; Jariwala, D.; Schuck, P. J. Facile and Quantitative Estimation of Strain in Nanobubbles with Arbitrary Symmetry in 2D Semiconductors Verified Using Hyperspectral Nano-Optical Imaging. *J. Chem. Phys.* **2020**, *153*, 024702.

(13) Schindelin, J. et al. Fiji: an Open-Source Platform for Biological-Image Analysis. *Nat. Methods* **2012**, *9*, 676–682.

(14) Florescu, M.; Torquato, S.; Steinhardt, P. J. Designer Disordered Materials With Large, Complete Photonic Band Gaps. *Proc. Natl. Acad. Sci. U. S. A.* **2009**, *106*, 20658–20663.

(15) Gorsky, S.; Britton, W. A.; Chen, Y.; Montaner, J.; Lenef, A.; Raukas, M.; Dal Negro, L. Engineered Hyperuniformity for Directional Light Extraction. *APL Photonics* **2019**, *4*, 110801.
